# Posner-Schlossman Syndrome

**DOI:** 10.7759/cureus.6584

**Published:** 2020-01-07

**Authors:** Jason Lippert, Michael Falgiani, Latha Ganti

**Affiliations:** 1 Emergency Medicine, Ocala Regional Medical Center, University of Central Florida College of Medicine, Ocala, USA; 2 Emergency Medicine, Envision Physician Services, Orlando, USA

**Keywords:** posner-schlossman syndrome, intraocular pressure, glaucomatocyclitic crisis

## Abstract

We present the case of a patient who presented to the Emergency Department with Posner-Schlossman syndrome, also known as glaucomatocyclitic crisis. While this condition is uncommon, it is essential that physicians be aware of its characteristics and how to treat it, otherwise affected patients are at risk of developing permanent complications such as optic nerve atrophy and loss of vision.

## Introduction

Posner-Schlossman syndrome (PSS), also known as glaucomatocyclitic crisis, is an ocular condition that presents acutely with markedly elevated intraocular pressure (IOP) and nongranulomatous anterior chamber inflammation [1]. It is most often classified as a secondary inflammatory glaucoma [1]. While the etiology is unknown [2], it is characterized by a decrease in vision, elevated IOP, open anterior chamber angles, and normal visual fields and optic nerve appearance [1].

On physical examination, PSS will generally present with unilateral blurred vision and mild eye discomfort or pain [3]. At times, the patient may not have any discomfort at all. An affected individual may also complain of halos instead of blurred vision. However, both are associated with corneal edema caused by an elevated IOP [3]. Patients may report a history of prior episodes; the length of these episodes and time in-between them is variable.

## Case presentation

A 79-year-old female presented to our Emergency Department (ED) with a chief complaint of blurred vision in her right eye prior to arrival. With this blurred vision, she reported seeing “flashes” and reported experiencing photophobia, pain with right-sided extra-ocular movement (EOM), and nausea with a single episode of vomiting. The patient was at church when these symptoms had suddenly begun. She had never experienced these symptoms before. The only relevant ophthalmic history the patient had was a history of cataract surgery.

The patient was hemodynamically stable, with initial vital signs including a heart rate of 64 beats per minute, a respiratory rate of 18 breaths per minute, and a blood pressure of 175/77 mmHg. On physical examination, the patient demonstrated bilaterally dilated and sluggish pupils, with painful right-sided EOMs, an injected right conjunctiva, and elevated IOP in both eyes on tonometry. The right eye IOP was 88 mmHg, and the left eye IOP was 75 mmHg. There was no temporal tenderness. She had no other significant examination findings.

The medical workup included electrocardiogram, basic metabolic panel, and complete blood count. No significant laboratory findings were found. The ophthalmology team was consulted. They recommended starting medications to decrease the IOP. Following these recommendations, we administered topical pilocarpine, timolol, and latanoprost drops, along with 500 mg of oral acetazolamide. The patient also received 1 liter of intravenous (IV) lactated ringers solution and 4 mg of IV ondansetron for nausea and vomiting.

Prior to administering mannitol, the ophthalmology team arrived and evaluated the patient at bedside. They confirmed that the patient did have an elevated IOP in the right eye, but upon examination found that the patient did not have an elevated IOP in the left eye. They also found that the patient had an open angle in the right eye and determined that the patient was suffering from PSS. They recommended that we still administer IV mannitol, with a goal of reducing the right eye IOP below 30 mmHg. They also advised that should the IOP not decrease sufficiently, topical 1% prednisone acetate be added for further control. A total of 250 mL of 20% IV mannitol was administered. The IOP did not improve with this treatment; therefore, topical prednisone was administered. Despite all of these measures, the patient maintained an IOP of 85 mmHg. Following this, we determined that the patient required overnight monitoring and care to prevent complications due to her condition. Our facility was not capable of monitoring the IOP overnight nor did they have sufficient capabilities for emergent surgery should it be required to relieve the patient’s IOP. Therefore, the patient was ultimately transferred to a higher level of care for her PSS.

## Discussion

PSS is a rare, typically unilateral and recurrent inflammatory ocular hypertensive disease [4]. It is classified as an inflammatory glaucoma because, by definition, it is always accompanied by uveitis [5]. The condition tends to affect patients between 20 and 50 years of age. While this condition is uncommon, it is essential that physicians be aware of its characteristics and how to treat it, otherwise affected patients are at risk of developing permanent complications from the disease, such as optic nerve atrophy and loss of vision [1].

PSS is believed to be caused by episodic changes in the trabecular meshwork, impeding outflow of aqueous humor, leading to an elevation of IOP (Figure 2) [1].

**Figure 1:**
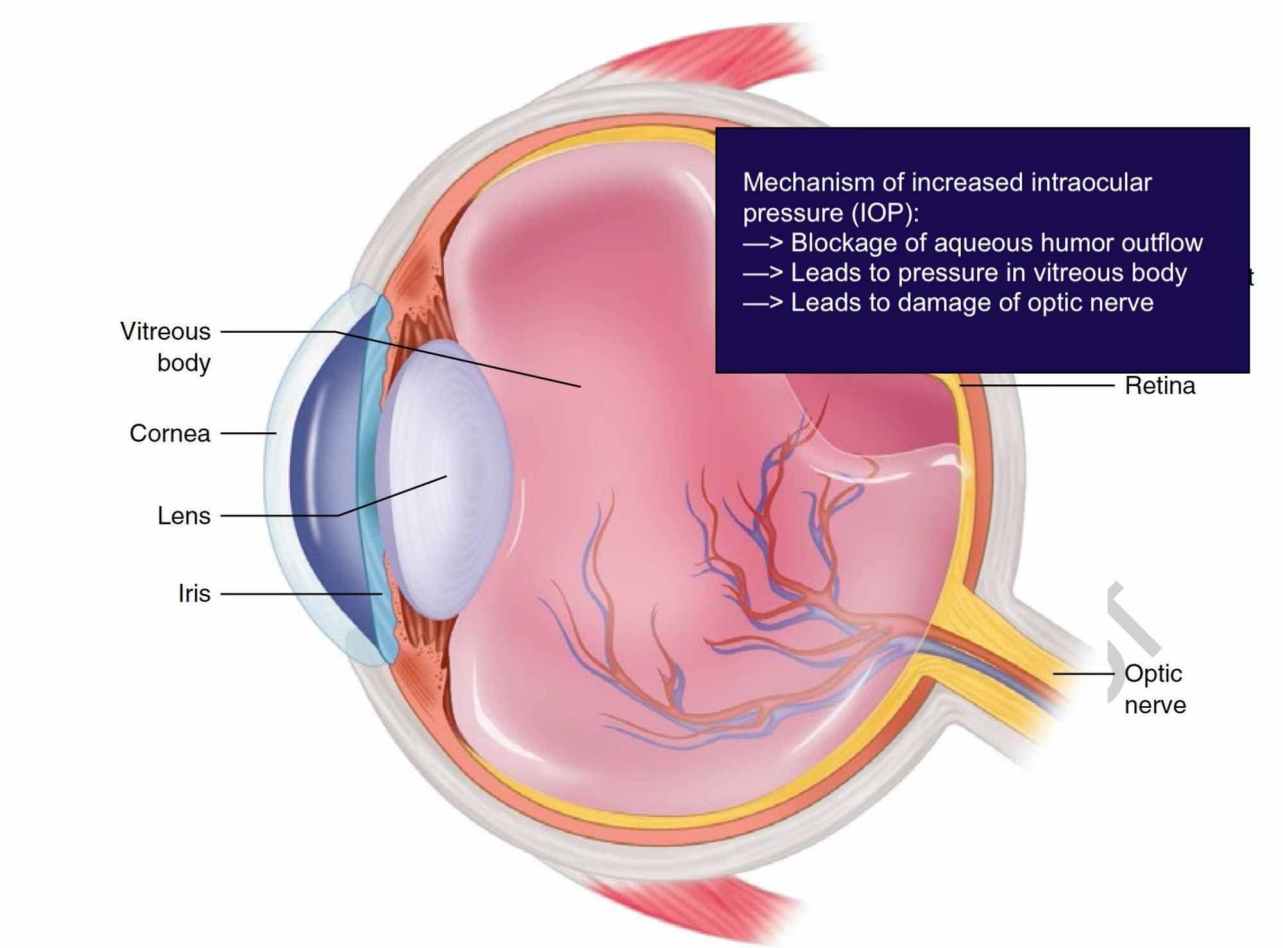
Eye schematic depicting the mechanism for the elevation of intraocular pressure Source: Modified with permission from Ganti [6]

This process is also accompanied by a mild intraocular inflammation [1]. However, despite this theory, there is not enough evidence to support this idea, and further research is required [3].

PSS patients will present with a history of unilateral blurred vision with or without eye discomfort or pain; however, there are reported cases of bilateral PSS [2]. Some patients also report seeing halos around lights. An affected individual’s vision will be determined by the level of associated corneal edema, ranging from visual acuity as high as 20/20 to vision being limited to light perception [2]. The pupil will often be slightly dilated and sluggishly reactive to light [2,7]. The conjunctiva may demonstrate no abnormalities or can demonstrate ciliary flush [2]. On slit-lamp examination, discrete, round, white keratic precipitates on the endothelium can be noted [2,4,7,8]. PSS will usually have significantly elevated IOP, potentially leading to corneal epithelial edema [1,2,4,7,8].

Treatment of PSS requires individualization of therapy and long-term management to prevent recurrence and complications from recurrence [1]. However, there is a lack of strong evidence that treatment prevents or reduces recurrence [4,8-9]. It has also been shown that PSS may resolve spontaneously within days to weeks, but despite this, treatment is still indicated to control IOP and inflammation [7]. Table 1 summarizes the drugs used in the management of elevated IOP.

**Table 1:** Drugs used to lower IOP IOP, intraocular pressure

Medication class	Examples	Administration route	Mechanism of action	Adverse effects
Prostaglandin analogs (first line)	Latanoprost, bimatoprost	Topical	Increase uveoscleral outflow	Conjunctival hyperemia, eye irritation, changes in the iris, and lash pigmentation
Carbonic anhydrase inhibitors	Acetazolamide, methazolamide	Oral or intravenous	Decrease aqueous humor production	Contraindicated in sulfa-allergic patients; not recommended in sickle cell patients
Alpha 2 agonists	Brimonidine	Topical	Decrease aqueous humor production	Conjunctivitis, hyperemia, ocular pruritus
Muscarinic agents	Pilocarpine	Topical	Increase aqueous humor outflow through the trabecular meshwork by the constricting ciliary muscles	Eye irritation, lacrimation, headache, blurry vision; not recommended in pregnancy
Hyperosmotic agents	Mannitol	Intravenous	Draws water out of (dehydrates) vitreous humor	Cerebral edema; caution with repeated doses
Beta-blockers	Timolol	Topical	Decrease aqueous humor production	Systemic absorption resulting in worsening congestive heart failure, airway resistance, and bradycardia in susceptible patients; reduce IOP less effectively than prostaglandin analogs

In addition, topical non-steroidal anti-inflammatory agents can be used to decrease the inflammation associated with PSS [1-2]. It should be noted that while these medications are useful for acute episodes of PSS, they have not been shown to reduce the recurrence of PSS [1,4]. Should medical treatment fail, surgical correction may be pursued, especially if there are signs of glaucomatous optic nerve damage, higher maximum IOP [2,10-11]. Surgical options include trabeculectomy, also known as filtering surgery [1-2,12-13].

## Conclusions

PSS, also known as glaucomatocyclitic crisis, typically presents as a unilateral eye complaint involving pain due to mild anterior uveitis, blurred vision, a lack of visual field defects, normal optic discs, and an open angle. The attacks tend to be recurrent. While PSS is not a common ED presentation, its acute presentation is usually due to elevated IOP, which is an ophthalmic emergency. Management centers on decreasing the IOP and inflammation.
